# The influence of service dog partnerships on perceived and objective sleep quality for military veterans with PTSD

**DOI:** 10.3389/frsle.2024.1432919

**Published:** 2024-09-04

**Authors:** Stephanie Bristol, Sarah C. Leighton, A. J. Schwichtenberg, Rebecca L. Campbell, Erin L. Ashbeck, Daniel J. Taylor, Edward J. Bedrick, Marguerite E. O'Haire

**Affiliations:** ^1^College of Veterinary Medicine, University of Arizona, Tucson, AZ, United States; ^2^Department of Psychology, College of Science, University of Arizona, Tucson, AZ, United States; ^3^Human Development and Family Science, Purdue University, West Lafayette, IN, United States; ^4^Statistics Consulting Lab, The BIO5 Institute, University of Arizona, Tucson, AZ, United States; ^5^Department of Epidemiology and Biostatistics, College of Public Health, University of Arizona, Tucson, AZ, United States

**Keywords:** actigraphy, complementary intervention, posttraumatic stress disorder, Assistance Dogs, service members

## Abstract

**Introduction:**

Sleep disturbances, fear of sleep, and nightmares are among the most reported symptoms for military service members and veterans (henceforth “veterans”) with posttraumatic stress disorder (PTSD), potentially contributing to treatment resistance and heightened suicidality. Despite available evidence-based interventions, many veterans still report difficulties. The complementary intervention of a psychiatric service dog may contribute to improvements in sleep quality for veterans with PTSD.

**Methods:**

This preregistered, non-randomized clinical trial evaluated the association between service dog partnership and sleep at 3 month follow-up. Participants were *N* = 155 veterans with PTSD (81 in the service dog group and 74 waitlisted controls). Measures included self-report surveys measuring PTSD severity (PTSD Checklist for DSM-5, PCL-5) sleep quality (Pittsburgh Sleep Quality Index, PSQI), sleep disturbance (PROMIS Sleep Disturbance), and fear of sleep (Fear of Sleep Inventory-Short Form, FoSI-SF); morning sleep diaries measuring nightmares; and wrist-worn actigraphy. Regression models and mediation analyses were performed.

**Results:**

Service dog partnership was significantly associated with better subjective sleep [PSQI: mean difference −2.2, 95% CI (−3.4, −1.1), *p* < 0.001; PROMIS Sleep Disturbance: −3.6, 95% CI (−6.3, −0.9), *p* = 0.004; FoSI-SF: −6.6, 95% CI (−9.7, −3.5), *p* < 0.001] and odds of nightmares [OR = 0.45, 95% CI (0.26, 0.76), *p* = 0.003]. Service dog partnership was not associated with a change in actigraphy-based measures of sleep [sleep onset: −4.5, 95% CI (−12.2, 3.2); sleep duration: −4.7, 95% CI (−25.9, 16.6); wake after sleep onset: 6.0, 95% CI (−2.1,14.1); sleep efficiency: −0.4, 95% CI (−3.4, 2.5)]. The estimated proportion of the effect of service dogs on PTSD severity mediated by fear of sleep was 0.26 [95% CI (0.10, 0.48)].

**Discussion:**

Compared to the control group, veterans with service dogs for PTSD reported better sleep quality, less sleep disturbance, and less fear of sleep after 3 months. However, service dog partnership was not associated with differences in actigraphy-measured sleep. These findings demonstrate the impact of psychiatric service dog partnerships on sleep quality for veterans with PTSD.

## 1 Introduction

Posttraumatic stress disorder (PTSD)—a mental health diagnosis resulting from trauma—is prevalent within military populations, with current lifetime estimates at 29% for post-9/11 veterans (U.S. Department of Veterans Affairs, 2023). Symptoms of PTSD include but are not limited to hyperarousal, flashbacks, intrusive thoughts, feelings of isolation, difficulty concentrating, and sleep challenges (American Psychiatric Association, 2013). Interestingly, sleep disturbances are often one of the earliest signs of a later PTSD diagnosis (Pace-Schott et al., 2015) and, along with nightmares, are frequently reported by military members and veterans (henceforth “veterans”) with PTSD (American Psychiatric Association, 2013).

While evidence-based treatment options for veterans with PTSD and comorbid sleep challenges may be effective, they are not palatable or efficacious for everyone (Bryant, 2019) and accessing care remains a challenge. Additionally, insomnia and nightmares have been shown to improve, but generally do not remit, after evidence-based PTSD treatment (Haynes et al., 2020; Larsen et al., 2019; Pruiksma et al., 2016; Schnurr and Lunney, 2019; Taylor et al., 2020). As many as 50–73% of service members continue to report nightmares, insomnia, or both following treatment (Pruiksma et al., 2016; Taylor et al., 2020). In addition, higher baseline insomnia and nightmare severity are associated with poorer PTSD treatment response (Belleville et al., 2011; Taylor et al., 2020). Shorter sleep duration, more insomnia symptoms, and worse self-reported sleep quality have been associated with increased PTSD symptoms the following day (Biggs et al., 2020; Short et al., 2017).

Complementary strategies to address sleep challenges for veterans with PTSD are needed. Psychiatric service dogs, trained in specific tasks to assist a person with a mental health diagnosis, are the third most common type of assistance dog worldwide (Assistance Dogs International, 2022). Service dog partnership has been associated with improved or lower PTSD symptom severity among military veterans with PTSD (Galsgaard and Eskelund, 2020; Jensen et al., 2021; Kloep et al., 2017; O'Haire and Rodriguez, 2018; Leighton et al., 2022, 2024). Moreover, veterans partnered with service dogs for PTSD self-report better sleep, both compared to veterans without a service dog and compared to their own sleep prior to service dog partnership (Kloep et al., 2017; McLaughlin and Hamilton, 2019; O'Haire and Rodriguez, 2018; Rodriguez et al., 2018). It is possible that fear of sleep could mediate the effect of service dog partnership on PTSD outcomes. Werner et al. (2021) suggests that fear of sleep develops from negative beliefs related to the loss of control during sleep, as well as fear of re-experiencing the traumatic event through nightmares. They propose that this leads to maladaptive behaviors such as delaying sleep or room checking at night, which can contribute to a sense of hyperarousal at bedtime. Service dogs are uniquely positioned to address this concern as their physical presence may be comforting to veterans and provide an increased sense of safety, reducing fear of sleep and hyperarousal. Additionally, if the veteran were to awaken following a nightmare, they are trained to assist with calming and comforting the veteran from anxiety. Finally, depending on their training, some service dogs are trained to interrupt nightmares by waking their handler. Thus, we propose that service dogs may reduce fear of sleep for veterans, which in turn contributes to improved sleep outcomes and decreased PTSD severity.

This study aimed to estimate the association between psychiatric service dogs and measures of subjective and objective sleep among post-9/11 military veterans with PTSD. We hypothesized that service dog partnership would be associated with (1) better sleep (reduced disturbances and improved overall sleep quality), via subjective sleep measures, (2) better sleep via objective (actigraphy-based) measures, and (3) reduced fear of sleep and nightmare symptoms. We also conducted an exploratory mediation analysis to (4) estimate the magnitude of the indirect effect of fear of sleep as a mediator of the effect of service dogs on PTSD severity.

## 2 Methods

### 2.1 Study design and participants

This article reports sleep outcomes from a pre-registered, non-randomized, independently monitored clinical trial (clinicaltrials.gov ID: NCT03245814). Ethical approvals were received from the Purdue University Human Research Protection Program Institutional Review Board (IRB protocol: 1702018766) and the Purdue University Institutional Animal Care and Use Committee (PACUC protocol: 1702001541). The trial was also monitored by an independent Data Safety and Monitoring Board (DSMB). Other previous publications have reported non-sleep-related outcomes (Jensen et al., 2022; Leighton et al., 2023, 2024; Nieforth et al., 2022a,b, 2023a, 2024) and sleep-related outcomes for spouses of veterans (Nieforth et al., 2023b).

Participants were recruited from a pool of military members and veterans with PTSD who had applied for and been approved to receive a service dog free of charge from the national non-profit organization K9s For Warriors (K9FW) between 2017 and 2020. Once added to the waitlist, participants were informed about the research study by members of the K9FW team. If interested in participating, veterans provided consent to share their contact information with the research team. All eligible veterans interested in participation received invitations, followed by a phone call from a member of the research team to review the materials and answer questions. If interested, the veteran provided verbal consent and scheduled screening activities, including (a) an online version of the informed consent document, (b) a short survey, and (c) verification of current PTSD diagnosis via blinded clinician assessment with the Clinical Administered PTSD Scale- 5 (CAPS-5) (Weathers et al., 2018).

Eligibility requirements for the study included a verified PTSD diagnosis, 18+ years of age, and K9FW eligibility criteria. K9FW specific eligibility criteria included (a) a completed written application, (b) reference forms, (c) interviews (d) community diagnosis of PTSD, military sexual trauma (MST), or traumatic brain injury (TBI), (e) no history of criminal behavior against animals (f) no more than two dogs in the home, (g) no current substance use, and (h) honorable discharge or current honorable service. If the veterans met these eligibility criteria, they were placed on a waitlist to receive a service dog (typically18–24 months) (K9s for Warriors, 2024).

Following consent and completion of screening activities, participants were assigned to a study group based on the K9FW scheduled date for their service dog partnership. The service dog group included participants who were scheduled to receive a service dog immediately following their 2-week baseline assessment, while the control group included participants who were still on the waitlist. All participants had unrestricted access to other forms of PTSD care and treatment. Study participation involved a baseline assessment and 3-month follow-up.

### 2.2 Service dog intervention

K9FW is an Assistance Dogs International-accredited non-profit service dog provider that trains psychiatric service dogs for active-duty military and veterans as well as station dogs for law enforcement and first responders. Potential psychiatric service dogs were selected from animal shelters, via owner relinquishment, or as puppies, and could be any breed, so long as they fit the size requirement (22–27 inches in height and < 80 pounds) and deemed temperamentally and medically suitable for a working role. During 60+ hours of training, service dogs received socialization and guidance in obedience and PTSD-related tasks. These tasks included interrupting anxiety, creating space between the veteran and others, and providing comfort following panic episodes. Once the dogs were deemed ready for partnership, the veteran visited a K9FW campus to take part in a 3-week training program where they learned how to work with their new service dog before returning home together. K9FW continued to support and stay in contact with each veteran-service dog team to provide additional follow-up guidance as needed for the duration of the partnership.

### 2.3 Measures

An online survey was administered at baseline and 3-month follow-up. Standardized self-report measures covered demographic information, PTSD symptom severity, insomnia symptoms, sleep-related impairment, fear of sleep, and sleep quality. Actigraphy monitoring was completed via a wrist worn Actiwatch 2.^1^ Participants also completed a morning sleep diary including questions about sleep and nightmares through a smartphone application on their personal cell phones.

#### 2.3.1 Demographics

Demographic surveys were developed and conducted using the Research Electronic Data Capture (REDCap) system (Harris et al., 2009). Self-reported demographic questions included race, gender identity, ethnicity, relationship status, education level, employment, number of children, number of pets in the home, history of deployment, and household income.

#### 2.3.2 Sleep disturbance

Sleep disturbance was assessed with the National Institute of Health (NIH) Patient Reported Outcomes Measurement Information System (PROMIS; Ader, 2007). This 8-item self-report measure collects information for the last seven days regarding nighttime sleep-related symptoms. Items include “My sleep was refreshing” or “I had a problem with my sleep”. Questions are presented on a 5-point scale and then converted to a standardized T-score following NIH PROMIS Sleep Disturbance guidelines.

#### 2.3.3 Sleep quality

The Pittsburgh Sleep Quality Index (PSQI) is a 19-item self-report measure that assesses the overall heuristic of sleep quality by collecting information on sleep environment, sleep disturbances, and daytime functioning (Buysse et al., 1989). A global score ranging from 0 to 21 is calculated from component scores with a score of 5 or above indicating poor sleep quality.

#### 2.3.4 Sleep diary

Prior-night sleep information was gathered through a 24-item sleep diary, administered through the LifeData RealLife Exp smartphone application for 14 days each at baseline and follow-up. Participants were prompted to complete the sleep diary upon awakening, with two reminders at 30-min intervals. The survey closed after 18 h. A total of 28 sleep diary entries were possible per participant, with one per day over 14 days at baseline and follow-up. Survey questions asked about the prior night, including co-sleeping, nightmares, awakenings, and how safe the participants felt as they were falling asleep. Outcomes utilized to assess trauma-related sleep disturbances were the veteran's sense of safety at night (“How safe did you feel as you were falling asleep?”), frequency of nightmares (“Did you have any nightmares or bad dreams last night?”) and the number of nightmare awakenings (“About how many times did you wake up because of a nightmare”). See Supplementary material S1 for the full list of questions included in the sleep diary.

#### 2.3.5 Actigraphy

Participants wore an Actiwatch 2 wristband (see text footnote^1^) for 14 consecutive 24-h days on their non-dominant wrists to track minute-by-minute movements. The band was water resistant, but participants were asked to remove it if swimming or bathing. Participants were instructed to press a button on the side of the wristband immediately before trying to sleep, immediately upon awakening, and to mark wristband removal (such as for swimming). After the assessment period, participants shipped the wristband to the research team for analysis. Actigraphy data were scored following gold standard methodology, with sleep and wake times informed by morning sleep diary responses and timing of button presses.

All nights of actigraphy data with acceptable levels of missingness (< 20% per nighttime sleep interval) were scored using the default algorithm within Actiware 6.1.2.1. Several previous studies also use this missingness standard (e.g., Abel et al., 2018; Schwichtenberg et al., 2016). The accelerometer sensitivity was set to 0.05 g-force with a bandwidth of 3–11 Hz. The sampling epoch was set at 1 min with one score per epoch. Rest intervals were set using the participant's daily sleep diary data. The auto rest interval feature was not utilized because initial data scoring checks revealed it was at times grossly inaccurate, especially for participants who followed atypical rest-activity patterns (i.e., shift work, long bouts of nighttime wakefulness). The sleep interval detection algorithm was set at 5 min of immobility. Functionally, this translated into 5 min with no activity triggering the start of their sleep interval (within their diary-established rest interval/their reported nighttime). A minimum of five consecutive 24-h periods of data were required for inclusion into the scoring protocol.

Actigraphy-based sleep measures included sleep duration (total sleep time), sleep onset latency (the time it takes to fall asleep when trying), wake after sleep onset (time spent awake after initially falling asleep), and overall sleep efficiency (percentage of time asleep during sleep window).

#### 2.3.6 Fear of sleep

The Fear of Sleep Inventory- Short Form (FoSI-SF) (Pruiksma et al., 2014) is a 13-item measure of fear of sleep in adults. A total calculated score ranges from 0 to 52 with higher scores indicating increased fear of sleep.

#### 2.3.7 Nightmares

Questions related to nightmares were included in the 24-item sleep diary (including “Did you have any nightmares last night?” and “Did your service dog wake you up while you were having a nightmare last night?”). See Supplementary material S1 for complete survey questions.

#### 2.3.8 PTSD

Participants completed the PTSD Checklist for DSM-5 (PCL-5), a self-report measure that uses 20 items to assess symptom severity (range 0–80, where a higher score indicates greater severity) (Weathers et al., 2013).

### 2.4 Analysis

Self-reported measures of sleep, including PSQI, PROMIS Sleep Disturbance, and FoSI-SF, were modeled with linear regression. The estimated difference in the mean outcome at follow-up between participants in the service dog group and the control group was adjusted for age, gender, baseline PCL-5 score, and the baseline value of the respective sleep measure.

Actigraphy-based sleep measures were modeled with linear mixed effects models, with random intercepts for participants. The estimated difference in the mean outcome at follow-up between participants in the service dog group and control group was adjusted for age, gender, baseline PCL-5 score, and the person-level mean sleep measure from the baseline assessment period.

Nightmares reported in sleep diaries during the follow-up assessment period were modeled with a generalized linear mixed effect model with logit link and random intercept for participant, to estimate the ratio of the odds of any nightmares, comparing participants in the service dog group with those in the control group. For the other sleep diary outcomes, including safety score and number of nightmare awakenings, linear mixed-effects models were used to estimate the difference in the mean between groups during the follow-up assessment period, with random intercept for participant. Model covariates included age, gender, baseline PCL-5 score, person-level mean sleep measure at baseline, and nightly bed-sharing.

**Figure 1:**
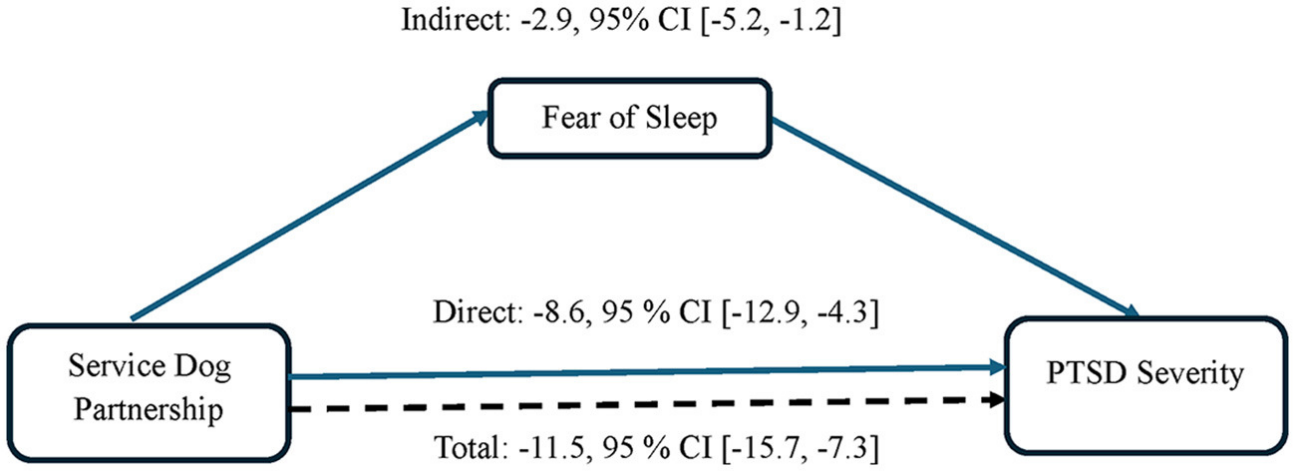
Impact of fear of sleep as a mediator of the effect of service dog partnership on PTSD severity.

To assess fear of sleep as a mediator of the effect of service dog partnership on PTSD severity, mediation analysis was performed under the counterfactual framework (Imai et al., 2010) (Figure 1). Linear regression was used to fit the mediator model for the conditional distribution of fear of sleep as measured by the FoSI-SF at follow-up, given treatment group (service dog vs. control) and covariates: baseline FoSI-SF, baseline PCL-5, gender, and age. The conditional distribution of PCL-5 scores at follow-up given the treatment group (service dog vs. control), FoSI-SF at follow-up, as well as the same covariates from the mediator model, was fit with linear regression. The average causal mediation effect (ACME) and average direct effect (ADE) (Tingley et al., 2014) were estimated and reported with non-parametric bootstrap bias-corrected and accelerated confidence intervals (Preacher and Hayes, 2008). Finally, we included an interaction between service dog and FoSI-SF in the model to consider a possible treatment-mediator interaction.

Given that some participants were lost to follow-up, did not complete follow-up sleep questionnaires, and/or did not complete baseline sleep questionnaires, analyses of the PSQI, PROMIS Sleep Disturbance, FoSI-SF, and mediation were conducted using multiple imputation to account for uncertainty about missing data values, with predictive mean matching. The imputation model included all variables in the analysis models as well as baseline variables associated with dropout (White et al., 2011).

Analyses were performed using R Statistical Software (v4.3.0) (R Team, 2023).

## 3 Results

The analysis used a modified-intention-to-treat (ITT) approach. Among *N* = 170 participants enrolled in the study, 10 were excluded from analysis in the service dog group because they did not receive a service dog during the study, and 4 were excluded from analysis in the waitlist group because they received a service dog prior to study follow-up assessment. One participant was excluded from analysis because they did not complete any sleep assessments at baseline or follow-up, leaving an analysis sample of *n* = 155.

In the modified ITT sample (*n* = 155), the extent of missingness at follow-up varied by outcome measure (PSQI, *n* = 23; PROMIS Sleep Disturbance, 22; FoSI-SF, 22; actigraphy-based outcome measures, 32; sleep diary outcomes, 22).

Participant characteristics are described in the service dog and control groups among 155 participants (Table 1). The average age of participants was 37.6 years (*SD* = 8.4) and most participants self-identified as male (75%), white (77%), and not Hispanic or Latino (78%). Most participants were married (63%) and not employed (65%). Participants reported an average of 3.3 household members (*SD* = 1.7), including a mean of 1.4 children living in the household (*SD* = 1.4), at least one pet dog (42%), and were making just enough income to make ends meet (52%). The majority (85%) of participants were deployed as part of their service and most (59%) were enlisted in the Army. At baseline, the overall mean PTSD severity score as measured by the PCL-5 was 56.5 (*SD* = 12.8).

**Table 1:** Baseline characteristics.

**Characteristic**	**Waitlist *n* = 74**	**Service dog *n* = 81**	**Overall *N* = 155**
Age, *M (SD)*	38.2 (8.5)	37.0 (8.2)	37.6 (8.4)
**Gender**			
Female	22 (30%)	17 (21%)	39 (25%)
Male	52 (70%)	64 (79%)	116 (75%)
**Race (*****n*** = **152)**, ***n*** **(%)**			
American Indian/Alaska Native	0 (0%)	0 (0%)	0 (0%)
Asian	2 (3%)	0 (0%)	2 (1%)
Black or African American	9 (12%)	8 (10%)	17 (11%)
Native Hawaiian or Other Pacific Islander	0 (0%)	2 (2.5%)	2 (1%)
White	55 (75%)	62 (78%)	117 (77%)
More Than One Race	5 (7%)	3 (4%)	8 (5%)
Prefer not to say	2 (3%)	4 (5%)	6 (4%)
**Ethnicity**			
Hispanic or Latino	14 (19%)	15 (19%)	29 (19%)
Not Hispanic or Latino	59 (80%)	62 (77%)	121 (78%)
Prefer not to say	1 (1%)	4 (5%)	5 (3%)
**Education (*****n*** = **154)**, ***n*** **(%)**			
Some high school	0 (0%)	0 (0%)	0 (0%)
High school/GED	4 (6%)	7 (9%)	11 (7%)
Some college	22 (30%)	32 (40%)	54 (35%)
2-year college degree	10 (14%)	14 (17%)	24 (16%)
4-year college degree	21 (29%)	17 (21%)	38 (25%)
Post-graduate degree	16 (22%)	11 (14%)	27 (18%)
Employed (*n* = 154), *n* (%)	26 (36%)	28 (35%)	54 (35%)
**Household income (*****n*** = **153)**, ***n*** **(%)**			
Comfortable	35 (48%)	27 (34%)	62 (41%)
Just enough to make ends meet	33 (45%)	46 (58%)	79 (52%)
Not enough to make ends meet	5 (7%)	7 (9%)	12 (8%)
**Relationship status (*****n*** = **155)**, ***n*** **(%)**			
Divorced	10 (14%)	12 (15%)	22 (14%)
Living with a significant other	4 (5%)	3 (4%)	7 (5%)
Married	52 (70%)	45 (56%)	97 (63%)
Separated	2 (3%)	7 (9%)	9 (6%)
Single	6 (8%)	14 (17%)	20 (13%)
Widowed	0 (0%)	0 (0%)	0 (0%)
**Household characteristics**			
Household members (*n* = 152), *M (SD)*	3.5 (1.7)	3.1 (1.6)	3.3 (1.7)
Number of children (*n* = 152), *M (SD)*	1.6 (1.4)	1.3 (1.4)	1.4 (1.4)
Pet dog (*n* = 153), *n* (%)	32 (44%)	32 (40%)	64 (42%)
**Bed partner or roommate (*****n*** = **152)**, ***n*** **(%)**			
No	18 (25%)	27 (34%)	45 (30%)
Partner/roommate in other room	9 (12%)	3 (4%)	12 (8%)
Partner/roommate in same room but not same bed	0 (0%)	0 (0%)	0 (0%)
Partner in same bed	46 (63%)	49 (62%)	95 (63%)
**Military characteristics**			
Deployed (*n* = 153), *n* (%)	59 (81%)	71 (89%)	130 (85%)
Military branch^*^(***n*** = **134)**, ***n*** **(%)**			
Air force	6 (11%)	5 (7%)	11 (8%)
Army	30 (53%)	49 (64%)	79 (59%)
Coast guard	2 (3%)	1 (1%)	3 (2%)
Marine corps	7 (12%)	15 (19%)	22 (16%)
National guard	3 (5%)	7 (9%)	10 (8%)
Navy	13 (23%)	5 (6%)	18 (13%)
**Mental health characteristics**			
PTSD Checklist (PCL-5), baseline, *M (SD)*	55.9 (14.3)	57.0 (11.3)	56.5 (12.8)
**Comorbid diagnoses (deployment-related)**			
Military sexual trauma (*n* = 153), *n* (%)	18 (25%)	19 (24%)	37 (24%)
Traumatic brain injury (*n* = 153), *n* (%)	36 (49%)	35 (44%)	71 (46%)
**Treatment**			
History of mental health treatment (*n* = 153), *n* (%)	38 (52%)	45 (56%)	83 (54%)
Concurrent mental health treatment (*n* = 153), *n* (%)	16 (22%)	23 (29%)	39 (25%)

^*^Percentages may be over 100% as some veterans were in multiple branches. PCL-5: PTSD Checklist for DSM-5.

### 3.1 Sleep disturbances and sleep quality

Service dog partnership was significantly associated with less sleep disturbance on the PROMIS Sleep Disturbance [difference in group means −3.6, 95% CI (−6.3, −0.9), *p* = 0.004] and better sleep quality on the PSQI [−2.2, 95% CI (−3.4, −1.1), *p* < 0.001] (Table 2).

**Table 2:** Sleep disturbance and sleep quality.

	**Baseline**, ***M*** **(SD)**		**Follow-up**, ***M*** **(SD)**			
	**Waitlist**	**Service dog**	**Waitlist**	**Service dog**	**Difference in group means (95% CI)**	***p***
PSQI	12.0 (4.4)	12.0 (4.4)	12.1 (4.2)	9.9 (4.8)	−2.2 (−3.4, −1.1)	< 0.001
PROMIS sleep disturbance	60.0 (7.1)	60.6 (8.0)	59.4 (9.0)	55.9 (9.5)	−3.6 (−6.3, −0.9)	0.004

PSQI, Pittsburgh Sleep Quality Index; PROMIS, NIH Patient Reported Outcomes Measurement Information System Sleep Disturbance. Raw scores were transformed to t-Scores (reported above) for the PROMIS Sleep Disturbance. Estimated difference in means at follow-up was adjusted for sleep measure at baseline, baseline PCL-5, age, and gender.

### 3.2 Actigraphy

Objective measures of sleep did not reflect significant differences between participants in the service dog and waitlist groups at follow-up (Table 3), including actigraphy-measured sleep duration [mean difference of −4.7 min, 95% CI (−25.9, 16.6), *p* = 0.66], sleep onset latency [−4.5 min, 95% CI (−12.2, 3.2), *p* = 0.25], wake after sleep onset [6.0 min, 95% CI (−2.1, 14.1), *p* = 0.15], and sleep efficiency [−0.4%, 95% CI (−3.4, 2.5), *p* = 0.76].

**Table 3:** Objective sleep quality.

	**Baseline**, ***M*** **(SD)**		**Follow-up**, ***M*** **(SD)**				
	**Waitlist**	**Service dog**	**Waitlist**	**Service dog**	**Difference in group means (95% CI)**		***p***
Sleep duration, min	384 (105)	376 (107)	390 (118)	381 (108)	−4.7	(−25.9, 16.6)	0.66
Sleep onset latency, min	21 (33)	20 (33)	24 (42)	19 (32)	−4.5	(−12.2, 3.2)	0.25
Wake after sleep onset, min	50 (30)	55 (38)	54 (35)	63 (47)	6.0	(−2.1, 14.1)	0.15
Sleep efficiency, %	81 (11)	80 (13)	79 (13)	78 (13)	−0.4	(−3.4, 2.5)	0.76

Sleep efficiency is measured on a 0–100 scale representing percent efficiency. Estimated differences in group means was adjusted for age, gender, baseline PCL-5 score, and mean sleep measure from the baseline assessment period. *P*-values were calculated from likelihood ratio tests.

### 3.3 Fear of sleep and nightmares

Participants in the service dog group reported lower fear of sleep on the FoSI-SF [mean difference −6.6, 95% CI (−9.7, −3.5), *p* < 0.001] and that they felt safer falling asleep compared to participants in the waitlist group [mean difference 0.7, 95% CI (0.4, 1.0), *p* < 0.001; Table 4]. The odds of nightmares at follow-up were significantly lower for participants in the service dog group compared to the control group [OR = 0.45, 95% CI (0.26, 0.76), *p* = 0.003]. The number of nightmare awakenings at follow-up also differed significantly by group [mean difference −0.1, 95% CI (−0.2, −0.0), *p* = 0.007] (Table 4).

**Table 4:** Trauma-related sleep disturbances.

	**Baseline**		**Follow-up**				
	**Waitlist**	**Service dog**	**Waitlist**	**Service dog**	**Difference in group means (95% CI)**		***p***
FoSI-SF	18.7 (10.8)	19.9 (12.9)	18.4 (12.1)	12.0 (11.4)	−6.6	(−9.7, −3.5)	< 0.001
Felt safe	3.9 (1.4)	3.6 (1.4)	3.8 (1.5)	4.3 (1.5)	0.7	(0.4, 1.0)	< 0.001
Number of nightmare awakenings	0.5 (1.7)	0.4 (0.9)	0.4 (1.0)	0.2 (0.6)	−0.1	(−0.2, −0.0)	0.007

FoSI-SF, Fear of Sleep Inventory-Short Form.

### 3.4 Impact of fear of sleep on PTSD severity

The estimated effect of a service dog on PTSD severity (PCL-5) mediated by fear of sleep (FoSI-SF) was −2.9 [95% CI: (−5.2, −1.2)], while the estimated total effect of a service dog on PTSD severity was −11.5 [95% CI: (−15.7, −7.3); Figure 1]. The estimated proportion of the effect of service dogs on PTSD severity mediated by fear of sleep was 0.26 [95% CI: (0.10, 0.48)] (Table 5). The treatment-mediator interaction between service dogs and FoSI-SF was not significant (*p* = 0.14).

**Table 5:** Mediation analysis: fear of sleep as a mediator of the effect of service dog on PTSD severity.

	**Estimate**	**95% CI**
Causal mediation effect	−2.9	(−5.2, −1.2)
Direct effect	−8.6	(−12.9, −4.3)
Total effect	−11.5	(−15.7, −7.3)
Proportion mediated	0.26	(0.10, 0.48)

## 4 Discussion

This study examined the effects of a psychiatric service dog on subjective and objective measures of sleep in post-9/11 veterans. Psychiatric service dog partnership was associated with higher self-reported sleep quality and less sleep disturbance after 3 months among post-9/11 veterans. Further, participants in the service dog group reported less fear of sleep and felt safer at night compared to participants on the waitlist. No significant differences in actigraphy-measured sleep were observed. Exploratory analyses suggested that fear of sleep partially mediated the association between service dog partnership and PTSD symptom severity, with fear of sleep accounting for ~26% of the average difference in PTSD symptoms. As reported in a separate publication, veterans in the service dog group reported significantly lower PTSD symptom severity on the PCL-5 (Leighton et al., 2024).

### 4.1 Veteran subjective sleep quality

The first aim of the study was to evaluate the impact of service dog partnerships on veteran's subjective sleep quality. As anticipated, subjective survey reports completed 3 months after service dog partnership showed fewer sleep disturbances and better self-reported sleep quality compared to waitlisted control participants.

Interestingly, previous self-report research on co-sleeping with pets has identified increased sleep disturbances due to animal noises during the night compared to those that sleep without pets (Smith et al., 2014). This differs from the current study which found lower self-reported nighttime disturbances in veterans with a service dog compared to the control group. This divergence may be explained by the substantial differences in pet ownership vs. service dog partnership. While pet dogs may bark or whine and be disruptive during the night, service dogs are trained not to whine or bark unless asked and may, therefore, be less likely to wake their owner.

An additional explanation for this difference relates to the most common PTSD symptom cluster, hyperarousal (Weston, 2014). Hyperarousal is a frequently reported symptom of PTSD and refers to a state of being constantly alert, making it difficult for the body to relax. This symptom is usually accompanied by an increased sympathetic response, also known as the “fight or flight” response, which can include feelings of panic and an elevated heart rate and blood pressure (Pole, 2007). Maintaining this heightened state of arousal impairs the body's ability to relax and may contribute to awakenings from small disturbances during the night. By partnering the veteran with a service dog, the veteran's sense of safety was impacted, potentially contributing to a decreased hyperarousal state during the night for the service dog group compared to those without a service dog. Future studies could examine this possibility by collecting biophysiological measures of hyperarousal at bedtime in comparison to a group without a service dog. Importantly, this difference in hyperarousal at night may persist whether the service dog is in the room with the veteran or elsewhere in the house. Future studies should further explore the impact of service dog proximity on sleep outcomes.

Evidence of greater self-reported sleep quality was found among veterans partnered with a service dog, and consistent with current literature examining the positive impacts of pets on self-reported sleep (Andre et al., 2021). This aligns with the finding of fewer reported disruptions, as better continuity of sleep lends itself to better overall sleep quality. This is a potentially important finding given that improved sleep has been suggested to improve PTSD treatment outcomes (Zalta et al., 2020).

### 4.2 Veteran objective sleep quality

The second aim of the study was to investigate objective sleep metrics as measured by actigraphy. No significant differences between veterans with and without service dogs were found for actigraphy-based measures of sleep duration, wake after sleep onset, sleep latency or sleep efficiency. These null findings fail to mirror our subjective findings of greater self-reported sleep quality. This is consistent with previous research on the impact of PTSD service dogs on veteran sleep (Lessard et al., 2020). Interestingly, this only partially aligns with research on pet dogs which has found that generally, sleeping with a pet improves self-reported sleep despite increases in objectively measured movement (Andre et al., 2021; Hoffman et al., 2020). With this finding, we contribute to a building body of literature that may call into question the utility of movement-based measurement when examining sleep quality. Actigraphy has varying levels of accuracy depending on individual characteristics such as gender and age (Danzig et al., 2020) and may lack sensitivity to change (Mitchell et al., 2019). Future studies may benefit from using polysomnography (PSG) as an objective measure because unlike the movement-based estimates of actigraphy, PSG uses multimodal assessment of brain activity, eye movement, and muscle tension to estimate sleep with high temporal accuracy.

Previous literature has noted increased objective movement during sleep when pets are present (Hoffman et al., 2020; Smith et al., 2018), though we did not observe significant differences in actigraphy-based outcomes.

One possible explanation includes differences in the populations studied. Much of the existing literature examined a sample comprised of mostly or entirely female adults (Hoffman et al., 2018; Smith et al., 2014, 2018) and previous research has found women to be more vulnerable to nighttime disturbances than men (Dittami et al., 2007). Given that 75% of our sample self-identified as male, this may be one explanation for the divergence of our results from previous studies. A second explanation for this difference is that the veterans in this study were already experiencing so many sleep disruptions, the addition of a service dog did not lead to further disruptions—effectively, a ceiling effect. While many studies focus on non-military civilians and their pets, our study examined a population of active-duty and military veterans with a history of traumatic events leading to PTSD diagnosis. Other pet owner studies to date have examined participants with relatively healthy sleep (Smith et al., 2014, 2018) whereas many of the veterans included in this sample were already experiencing disrupted sleep.

Finally, it is also possible that for veterans with PTSD, service dog partnership impacts areas of sleep unrelated to motion and therefore not possible to measure through actigraphy.

### 4.3 Fear of sleep and nightmares

Our third aim was to investigate whether service dog partnership reduced fear of sleep and nightmare symptoms.

Veterans with service dogs reported less fear of sleep and higher feelings of safety while falling asleep. This is consistent with companion animal research, where owners report feeling safer at night with their pet dog present (Smith et al., 2018). Previous research has shown that fear of sleep is commonly reported in those with PTSD and has a direct relationship with PTSD severity (Kanady et al., 2018). Two common explanations for fear of sleep in veterans are (1) loss of vigilance and (2) nightmares.

Like hyperarousal, vigilance involves frequent checks of one's surroundings to observe for any signs of potential danger (Hull et al., 2016). While on duty, military members are often guarded by other service members at gates and entrances. The transition home can be difficult when this sense of security suddenly disappears. Concerns over losing vigilance at night leads many veterans to fear going to sleep, which impacts overall sleep outcomes (Pietrzak and Southwick, 2010). By having a service dog present that can alert the veteran throughout the night if needed, the veteran's fear of letting their guard down was likely reduced, allowing them to sleep more soundly.

The second reason veterans often fear sleep is due to the prevalence of recurring, vivid nightmares. Importantly, veterans may experience specific nightmares related to the traumatic experience (i.e., combat) such as replaying the trauma or a threat to their life (Esposito et al., 1999; Khazaie et al., 2016). Due to the recurrent and challenging nature of these nightmares, veterans often feel anticipatory anxiety before going to bed. They may delay sleep for as long as possible or use alcohol or drugs to cope (Hughes et al., 2018). While these strategies may reduce anxiety in the short-term, these maladaptive coping strategies can, in turn, make nightmares more intense or more frequent.

This study found lower odds of nightmares in veterans with service dogs compared to those on the waitlist. This is consistent with qualitative reports of decreased nightmare frequency and intensity with a service dog present (Scotland-Coogan, 2019). This may be due to the potential reduction in hypervigilance with the service dog present, the ability of the dog to comfort the veteran following a nightmare, or the ability of some service dogs to recognize signs of physical distress and interrupt nightmares in their veteran partners, pulling them out of the experience (Rodriguez et al., 2020).

### 4.4 Impact of fear of sleep on overall PTSD severity

We conducted an exploratory analysis of fear of sleep as a potential mediator of the association between service dog partnership and PTSD severity. We estimated that the indirect effect of service dogs via less fear of sleep accounted for 26% (95% CI: 10%, 48%) of the difference in PTSD severity scores. Given that fear of sleep manifests as hyperarousal, sleep outcomes would naturally be affected (Altena et al., 2017; Kalmbach et al., 2018; van Wyk et al., 2016; Werner et al., 2021). By addressing these factors, partnership with a service dog for PTSD may reduce barriers to sleep, in turn lessening overall symptom severity.

This finding points to one of the potential mechanisms through which service dogs may impact PTSD outcomes. Given that this pathway through fear of sleep accounted for less than half of the difference in PTSD severity, it may act in concert with other mechanisms that have been proposed. For example, prior research has suggested that the service dogs' physical presence (Leighton et al., 2023), trained task usage (Jensen et al., 2022; Leighton et al., 2023) and impact on stress hormones (Nieforth et al., 2024) may underly the impact of service dog partnership on PTSD severity. In combination, we begin to develop a more complete picture of the mechanisms through which service dogs may benefit veterans with PTSD. However, it is important to note that service dog partnerships should not be considered a replacement for trauma-focused therapy, but rather a complementary intervention that can contribute to improved PTSD outcomes.

Research has also not yet determined the degree to which service dogs could be considered a safety behavior for PTSD (Leighton et al., 2022). Veterans in this study had lower fear of sleep in the service dog group, which could represent either an underlying belief that their service dog is keeping them safe from external threats *or* that they receive supporting comfort to attune their physiological regulation to a calming external presence (National Academies of Sciences, 2021). For the former, it should be noted that service dogs are not (and should not be) trained in guarding behaviors (Assistance Dogs International, 2024). For the latter, if veterans recognize that there is no real threat and that the dog's presence is intended to help regulate their emotions and calm them at night, service dogs would not be considered a safety behavior. Thus, an important direction for future research will be to investigate not only the extent to which service dogs can help with sleep but also veterans' underlying beliefs about a service dog's role in helping with PTSD. In turn, this information could guide service dog organizations in maximizing client education and service dog training effectiveness.

## 5 Limitations

There are important limitations to this study. First, participants were not randomly assigned to the service dog and waitlisted control groups. Instead, they were allocated based on their position on the waitlist. Second, this study worked with a single service dog provider whose selection criteria or application process may limit the generalizability of our findings to the broader population of veterans with PTSD. Furthermore, different service dog provider organizations may train different tasks (including nightmare interruption) or have other variations in their procedures that could differentially impact sleep outcomes for their clients. Third, other potential confounding factors, such as time since the traumatic events, medication usage, and past or current PTSD therapy, were not accounted for in the present analysis. Lastly, participants were only followed for three months after service dog placement.

## 6 Conclusion

Findings from this study demonstrate the potential impact of service dog partnerships on veteran sleep, and may reflect a mechanism by which service dog partnership reduces PTSD severity. Results indicate significant differences in subjective, but not objective measures of sleep, illustrating the potential influence service dogs have on aspects of sleep unrelated to movement. These outcomes highlight the importance of utilizing multiple data collection methods when attempting to understand sleep-related functional impairments. Exploratory mediation results suggest that decreased fear of sleep may partially explain how service dog partnership leads to improved PTSD outcomes, offering insight into one of the potential mechanisms through which service dogs may impact overall PTSD severity. These findings build on existing sleep literature and demonstrate the potential impact of service dog partnerships on veteran sleep and PTSD outcomes.

## Data Availability

The datasets presented in this article are not readily available because of confidentiality concerns. Deidentified data is available upon reasonable request to the corresponding author. Requests to access the datasets should be directed to Marguerite E. O'Haire, maggieohaire@arizona.edu.

## Footnotes

^1^Philips Respironics. https://www.usa.philips.com/healthcare/product/HC1044809/actiwatch-2-activity-monitor.
