# Supplementary material for: The influence of service dog partnerships on perceived and objective sleep quality for military veterans with PTSD
Source: Front Sleep. 2024 Sep 4;3:1432919. doi: 10.3389/frsle.2024.1432919 (PMC12713846; doi:10.3389/frsle.2024.1432919)
Supplement: Supplementary file 1 [file Table_1.DOCX]

Supplementary Material

# S1: Morning Sleep Diary Questions

1. Did you take any naps yesterday?

◯ Yes

◯ No

1. How long did you nap yesterday? *HH:MM:SS*
2. What time did you lay down to go to sleep last night? *Date,* *HH:MM:SS*
3. How safe did you feel as you were falling asleep?

◯ Not at all ◯ Moderately ◯ Very Much

◯ Slightly ◯ Quite a bit ◯ An extreme amount

◯ Somewhat

1. What time did you wake up for the day today? *Date,* *HH:MM:SS*
2. Did anyone else sleep in your bed last night (people or animals)?

◯ Yes

◯ No

1. (If yes to 6) Who slept in your bed last night? (Select all that apply)

☐ Pet dog ☐ Pet cat ☐ Child

☐ Service dog ☐ Adult

1. (If yes to 6) Were these individuals or animals in your bed for

◯ All of the night ◯ Second half of the night

◯ First half of the night ◯ Only a small portion of the night

1. (Service dog group at follow-up only) When was the service dog *in the bed* with you? (Select all that apply.)

☐ When I got into bed ☐ When I first opened my eyes this morning ☐ When I fell asleep ☐ When I first got out of bed this morning

☐ When I woke up in the night ☐ None of the above

1. (Service dog group at follow-up only) When was the service dog *in the room* with you (but *not* in the bed)? (Select all that apply.)

☐ When I got into bed ☐ When I first opened my eyes this morning ☐ When I fell asleep ☐ When I first got out of bed this morning

☐ When I woke up in the night ☐ None of the above

1. Did you have any nightmares or bad dreams last night?

◯ Yes

◯ No

1. (If yes to 11) How upsetting were the nightmares?

◯ Not at all ◯ Moderately ◯ Very Much

◯ Slightly ◯ Quite a bit ◯ An extreme amount

◯ Somewhat

1. (If yes to 11) What were your nightmares related to?

◯ A traumatic event

◯ Other

◯ I don’t remember

1. Do you remember waking up from the nightmares?

◯ Yes

◯ No

1. (If yes to 14) About how many times did you wake up because of a nightmare?
   1. Selects 1-30 on a visual number wheel
2. (Service dog group at follow-up only & if yes to 14) Did your service dog wake you up while you were having a nightmare last night?

◯ Yes

◯ No

1. (If yes to 14) Were you able to fall back asleep after waking from the nightmares?

◯ Yes

◯ No

1. (If yes to 17) For your longest nightmare waking, how long did it take you to fall back asleep?
   1. Amount of time
2. Do you remember waking up at all from other things while sleeping last night? (not from nightmares)?

◯ Yes

◯ No

1. (If yes to 19) Why did you wake up? (Select all that apply.)

☐ Startle or panic-like awakening without remembering a dream

☐ Being too hot or too cold

☐ Bodily functions (needing to use bathroom, coughing, snoring, etc.)

☐ Dog disturbance (movement or snoring)

☐ Human disturbance (spouse or child snoring, moving around)

☐ Disturbance unrelated to bedmates (storm, text message, noise outside)

☐ Tossing and turning, couldn’t get comfortable

☐ Unknown or other reason

1. (For any selected in 20) About how many times did you wake up because of <<insert item from 20>>?
   1. 1-30 (number wheel)
2. (If yes to 19) Were you able to fall back asleep after waking?

◯ Yes

◯ No

1. (If yes to 22) For your longest waking not caused by nightmare, how long did it take you to fall back asleep?
   1. Amount of time
2. Is there anything else you would like to share (optional)?
   1. Free response

**Note*  Red text indicates questions or answer choices that are only given at follow-up to those who have received a service dog
